# GIP as a Therapeutic Target in Diabetes and Obesity: Insight From Incretin Co-agonists

**DOI:** 10.1210/clinem/dgaa327

**Published:** 2020-05-27

**Authors:** Jens Juul Holst, Mette Marie Rosenkilde

**Affiliations:** 1 Department of Biomedical Sciences, University of Copenhagen, Copenhagen, Denmark; 2 NNF Center for Basic Metabolic Research, University of Copenhagen, Copenhagen, Denmark

**Keywords:** GLP-1, type 2 diabetes, weight-losing therapy, glucose-dependent insulinotropic polypeptide, receptor internalization, co-agonists

## Abstract

The 2 hormones responsible for the amplification of insulin secretion after oral as opposed to intravenous nutrient administration are the gut peptides, glucagon-like peptide-1 (GLP-1) and glucose-dependent insulinotropic polypeptide (GIP). However, whereas GLP-1 also inhibits appetite and food intake and improves glucose regulation in patients with type 2 diabetes (T2DM), GIP seems to be devoid of these activities, although the 2 hormones as well as their receptors are highly related. In fact, numerous studies have suggested that GIP may promote obesity. However, chimeric peptides, combining elements of both peptides and capable of activating both receptors, have recently been demonstrated to have remarkable weight-losing and glucose-lowering efficacy in obese individuals with T2DM. At the same time, antagonists of the GIP receptor have been reported to reduce weight gain/cause weight loss in experimental animals including nonhuman primates. This suggests that both agonists and antagonist of the GIP receptor should be useful, at least for weight-losing therapy. How is this possible? We here review recent experimental evidence that agonist-induced internalization of the two receptors differs markedly and that modifications of the ligand structures, as in co-agonists, profoundly influence these cellular processes and may explain that an antagonist may activate while an agonist may block receptor signaling.

The incretin hormones are normally responsible for a very large part of the postprandial insulin secretion and therefore for postprandial glucose tolerance—their contribution (via insulin secretion) to postprandial glucose clearance may be as great as to correspond to 80% of the ingested amount of glucose (1). The incretin effect is almost completely lost in people with type 2 diabetes (T2DM) and restoration of this deficiency would therefore be of great interest (2,3). Two hormones, glucagon-like peptide-1 (GLP-1) and glucose-dependent insulinotropic polypeptide (GIP), are normally responsible for the effect, but infusion studies have shown that only 1 of them, GLP-1, is capable of stimulating insulin secretion in patients with T2DM, while GIP is almost inactive (4). Another important difference is that GLP-1 inhibits appetite and food intake (5), resulting in weight loss upon chronic administration, whereas GIP generally is thought to have no effects on food intake (6). Despite this, some researchers have kept working with GIP and have developed analogs, modified to have activity also on other receptors including the GLP-1 receptor. Application of these in clinical trials has been surprisingly successful. Most recently, the pharmaceutical company, Eli Lilly, presented impressive Phase 2 results in overweight patients with T2DM treated with tirzepatide, a mono-molecular, long-acting (weekly) GIP-GLP-1 co-agonist (7). A 6-month treatment with this compound resulted in near-normalization of glycated hemoglobin levels and weight losses reaching 2-digit percentages, with both effects exceeding what was obtained in the same study with a long-acting GLP-1 receptor agonist, dulaglutide. Increasing the confusion even further, another company, Amgen, presented preclinical data from nonhuman primates showing that a GIP receptor antagonist (a monoclonal antibody), both alone and in combination with GLP-1, effectively reduced the normal increase in body weight in obese animals (8). In addition, upon comparison of results from animal studies, it appears that almost identical results can be obtained with certain proven GIP agonists and GIP antagonists, at least with respect to their effects on body weight (8-10). In other words, both GIP agonism (normally thought to be inactive) and GIP antagonism appear to be effective in T2DM and obesity. How is this possible?

The full answer to this question cannot be given presently, but it appears that the manipulations with the agonist structure may have consequences that reach beyond the simple key-and-lock activation of its cognate receptor. This could happen if the manipulated co-agonist activates a different set of pathways compared to the endogenous agonists (also known as biased agonism). It should also be considered that a combined activation of the GIP as well as the GLP-1 system could result in beneficial effects beyond those obtained by a simple addition of the 2 separate effects, either by concomitant or by sequential activation of the 2 hormone systems.

GIP, a 42-amino acid polypeptide secreted from endocrine K-cells of the upper small intestinal epithelium, was the first incretin to be established (11,12), and in careful mimicry studies, Nauck et al (13) demonstrated that the insulinotropic effects of GIP infusions, resulting in plasma concentrations similar to those observed after oral glucose ingestion, could fully explain the insulin response to oral glucose. The insulinotropic effects of the other incretin hormone GLP-1 (14,15) and its possible incretin role (16), were described 1987. Again, in accurate mimicry studies, it was demonstrated that both GIP and GLP-1, infused to plasma concentrations precisely mimicking postprandial concentrations, would stimulate insulin secretion, about equally, in a glucose dependent manner; weakly at fasting glucose levels and more powerfully at higher (still normal) postprandial levels (17). Around 1992, Raufman and Eng in New York characterized the Gila monster peptide exendin-4 (18), which turned out to be a full agonist for the GLP-1 receptor (19) (a synthetic version of which, exenatide, reached the market in 2005 (20)). Building on experience from their isolation of the related peptide, exendin-3, they also identified a powerful and seemingly specific GLP-1 receptor antagonist, namely, the truncated peptide, exendin 9-39 (18). With this tool, which was administered to humans in 1998-1999 (21), it was possible to characterize the actions of endogenous GLP-1, and in several studies (22,23), the insulinotropic actions of endogenous GLP-1 were confirmed using exendin 9-39, which typically reduced insulin responses to oral/intestinal glucose administration in humans. For GIP, an effective and potent receptor antagonist for human use was only recently introduced (24), but with these tools at hand, it was now possible to analyze the combined actions of GIP and GLP-1 in the same experiment. Thus, in healthy subjects, infusions of each of the 2 antagonists reduced impaired oral glucose tolerance and reduced glucose induced insulin secretion. In combination, they clearly had additive effects (25). It was calculated that whereas glucose alone was responsible for 33% of the insulin response to oral glucose, GIP was responsible for 44% and GLP-1 for 22% of the response (26). While largely confirming the mimicry experiments, the use of exendin 9-39 is not unproblematic, because the antagonist also greatly increases plasma glucose and glucagon levels, which complicates the interpretation considerably (27).

As already alluded to, infusions of GLP-1 in supraphysiological amounts or administration of GLP-1 receptor agonists are capable of inducing insulin secretion and appetite reduction also in obese patients with T2DM, whereas infusions of GIP are remarkably ineffective, regardless of infusion rate (28). Also when infused together, only the GLP-1 part has apparent effects on insulin secretion and blood glucose. In fact, in an experiment with co-infusion, the suppression of glucagon secretion by GLP-1 was obliterated by co-infusion with GIP, perhaps in agreement with the observation that GIP, if anything, stimulates glucagon secretion, particularly in T2DM patients, an effect that might actually contribute to the development of hyperglycemia (29). In further studies of appetite and food intake, infusions of GLP-1 increased insulin secretion and inhibited food intake, whereas infusions of pharmacological amounts of GIP were ineffective and even appeared to prevent the inhibitory effects of GLP-1 on food intake (30). A similar finding was made in experiments in which GLP-1 agonism had been maintained chronically, namely, in patients with T2DM during stable therapy with the GLP-1 RA, liraglutide. In these patients, infusions of pharmacological amounts of GIP increased glucagon concentrations, impaired postprandial lipids, and increased postprandial glycemia but had no effect on food intake (31). In view of these studies and many more, which all consistently have demonstrated lack of GIP efficacy in T2DM, the results obtained with the new GIP-GLP-1 co-agonists seem incomprehensible.

The inactivity of GIP in T2DM has been investigated in experimental animal models, and it has been reported that hyperglycemia reduces GIP receptor expression in the beta cells and that treatment of the hyperglycemia restores GIP receptor expression and beta cell responsiveness (32). In studies of individuals with long-standing T2DM, in whom physiological GIP infusions were completely without effect, a 4-week period of intensive, basal-bolus insulin therapy, which nearly normalized glucose levels, there was some restoration of GIP’s insulinotropic effect, but the responses were still very far from normal levels or levels observed after pharmacological GLP-1 therapy (33). In addition, during clamp studies in patients with T2DM (28), a small early insulin response may actually be induced with high dose GIP infusions (whereas the second-phase response is completely absent), and this early response is, although smaller than that observed in controls, impaired to the same extent as the early response to GLP-1. This suggests that the overall loss of the insulinotropic of GIP in T2DM is due to postreceptor defects associated with prolonged beta cell stimulation, rather than to a specific, glucose-induced down-regulation of the GIP receptor (34).

Upon further analysis of co-administrations of the GLP-1 and GIP antagonists under various circumstances, additional interesting features have emerged. In healthy individuals, GIP has effects other than stimulating insulin secretion. GIP is probably one of the important actors in the so-called gut-bone axis, a term introduced to describe the 50% reduction in bone resorption (as measured by bone resorption markers; eg, C-terminal telopeptide of type 1 collagen) that occurs after food intake, compared to the fasting rate (35). Thus, during most of the daytime, bone resorption is reduced, while a corresponding increase is observed during the night time, whereby a constant bone mass is maintained. It turns out that GIP infusions in humans are capable of causing similar reductions in bone resorption (36). The effect is apparently due to a direct effect of the hormone on GIP receptors expressed on both osteoblasts and osteoclasts, the functions of which are, respectively, enhanced and inhibited. In agreement with the supposed actions of GIP, administration of the GIP antagonist GIP (3–29) NH_2_ greatly reduced the meal-induced suppression of bone resorption, and these experiments thus confirmed the important contribution of GIP to the gut-bone axis (37). In further studies, it turned out that administration of the same GIP antagonist markedly reduced the meal-induced bone resorption, even in individuals with T2DM. First, this indicates that the GIP part of the gut-bone axis is also operative in these patients, and second, it suggests that GIP receptor expression and function in the bone cells is not affected in T2DM (38). It can therefore be concluded that if a change in GIP receptor expression or function is involved in the impaired insulin response to GIP in T2DM, this change is likely to be relevant only for GIP receptors expressed in beta cells.

The studies supporting antidiabetic and weight-reducing actions of GIP and GIP co-agonists date back to an early study in rodents with a monomolecular GIP-GLP-1 co-agonist, which was found to both enhance glucose tolerance and to lower body weight (39). This was of cause unexpected since GIP in humans, as previously discussed, exerted opposite effects in combinations with GLP-1 infusions. In fact, GIP had for a long time and for many reasons been considered “the obesity hormone” (40); for instance, its secretion is enhanced by intake of fatty meals, and GIP infusions in experimental animals were reported to enhance chylomicron clearance and fat deposition (41). Indeed, in 2002, mice with a knockout of the GIP receptor were demonstrated to be resistant to the adipogenic effect of a high-fat diet (42), and human genetic studies identified inactivating (Rosenkilde et al, unpublished) mutations in the GIP receptor, which were associated with weight loss (43). Altogether, rather than promoting weight loss, it was anticipated that GIP actions would promote weight gain and that a rational approach to obesity therapy therefore might be application of a GIP antagonist. Indeed, GIP antagonism in the form of a monoclonal antibody against the GIP recepto*r* turned out to be effective with respect to inhibiting food intake and promoting a weight loss in both rodents and in obese nonhuman primates (8). However, what was clearly missing in the human studies was a long-acting GIP antagonist, and there are still no data available regarding long-term actions of GIP agonism in humans. In rodents, however, long-acting GIP agonists with an improved design were recently reported to have in weight losing properties (44), and in the same series of studies long-acting (acylated) GIP antagonists did not cause weight loss in diet-induced obese animals. Furthermore, recent elegant studies suggested that certain somatostatinergic neurons in the rodent hypothalamus express GIP receptors and react to activation of these by decreasing food intake (45). These newer findings raise the question whether there are species differences regarding the effects of GIP on appetite and food intake.

Currently, therefore, we have two opposing viewpoints, one maintaining that GIP antagonism would be beneficial with respect to at least weight management and the other proposing that GIP agonism, perhaps preferably in conjunction with GLP-1 agonism, would be effective.

## Is It at All Possible to Reconcile the Two Viewpoints?

The people behind the development of the GIP receptor antibody have looked at the possible mechanisms (10) and focused on GIP receptor down regulation. It is known that GIP activation of its receptor is associated with recruitment of beta arrestins and that arrestins are needed for the subsequent internalization of the hormone receptor complex (46). By extended exposure of a GIP receptor expressing tissue to GIP, it would therefore be possible to create profound down regulation and therefore desensitization of the GIP receptor and impairment of the GIP sensitivity of the tissue. Indeed, this was directly demonstrated by Mohammad et al (47), who showed that an initial GIP stimulation can impair subsequent GIP stimulations, associated with disappearance of GIPR from the plasma membrane in 3T3-L1 adipocytes. This mechanism would be consistent with the remarkable lack of responses to increasing GIP concentrations, brought about by infusions of GIP, on top of the normal meal responses in healthy subjects (6). Furthermore, it was recently shown that the GIP receptor antagonist GIP (3–29)NH2 was able to restore the cell surface expression of the GIP receptor in transfected HEK293 cells after pre-incubation (and thereby agonist-induced receptor internalization) with endogenous GIP (46). Hence, it may be anticipated that antagonizing endogenous GIP actions in vivo, as can be done with both receptor antibodies and with peptide-based GIP receptor antagonists including GIP (3-29)NH2 in humans, would result in increased receptor expression on the cell surface, whereby the sensitivity of the system is regained. It is, however, still difficult to understand how GIP can activate the receptor in the presence of an antagonist, given the competitive nature of at least peptide-based GIPR antagonists (48). Nevertheless, the receptor internalization process is apparently important for GIP actions. For instance, when studied in vitro, the well-known GIP receptor mutation E354Q, which is associated with impaired glucose tolerance and increased fracture risk in postmenopausal women (49), actually shows enhanced agonist-mediated and basal 3′,5′-cyclic AMP formation and maintained arrestin recruitment, but prolonged agonist residence time, resulting in accelerated internalization and therefore impaired overall activation of the receptor signaling (50,51). This mutation is also associated with a slower recycling of internalized receptors to the cell surface, which, although it has been shown that the GIP receptor may also signal from endosomes (52), probably contributes to an overall impaired receptor function.

Thus, an effect on receptor recycling is apparently important for the actions of both GIP agonists and antagonists. But what about the effects of the GIP-GLP-1 co-agonists and their apparently beneficial metabolic actions? As previously discussed, the beneficial effect of GIP receptor activation is difficult to understand, as the effect of GIP is impaired in patients suffering from T2DM and obesity. So how can a dual-acting GIP-GLP-1 receptor agonist be better than the GLP-1 part of the combination? At first, it might be considered whether this is indeed the case. Upon closer scrutiny, the first dual GIP-GLP-1 co-agonist (NN9709, formerly MAR709 and RG7697) wasn’t terribly impressive after all, and its performance in a Phase 2 clinical trial did not differ from that of liraglutide (53). The second, tirzepatide, was clearly superior to the GLP-1 RA control, dulaglutide, in the dose-finding Phase 2 study mentioned in the beginning (7) although it was not ensured that optimal dosing had been investigated for the comparator—the fact that increasing doses of dulaglutide are currently being investigated (54) might suggest that the dose employed in the Phase 2 study was suboptimal. Nevertheless, as already mentioned, it is possible that the administration of a molecule that can activate both the GIP and the GLP-1 receptor may be beneficial in a sequential manner. Thus, the activation of the GLP-1 system might be the primary beneficial action, so that the beneficial effect of GIP may only be observed after metabolic control has been (partly) restored by GLP-1. In other words, the insulinotropic action of GIP may be regained after a GLP-1-mediated lowering of the blood glucose in agreement with the beneficial effects of intensive insulin therapy as previously mentioned (33). However, the disappointing results of adding high-dose GIP infusions to chronic liraglutide treatment (31) speak against this possibility. Another explanation could lie in a different pharmacodynamic profile of the dual agonist as compared to the individual signaling profiles of GIP and GLP-1, for instance caused by altered signaling of 1 or both of the 2 components. In fact, it has been shown that even small changes in the GIP as well as the GLP-1 molecule may change the receptor signaling towards a preferential G protein signaling with decreased arrestin recruitment and/or reduced receptor internalization (for GIP changes, see (51); for GLP-1 changes, (55, 56)). For the GIP system, such an effect would be beneficial due to a lower degree of receptor desensitization and internalization, and thereby improved therapeutic effect, given the proven downregulation of this system upon prolonged GIP administration (47,51,50). For the GLP1-1 system, receptor internalization seems independent of arrestin recruitment (57). Nevertheless, it was recently shown that N-terminal modifications of exendin-4 (a strong GLP-1 receptor agonist) will turn it into a biased agonist with a lower tendency to arrestin recruitment and/or receptor internalization and therefore with potentially greater efficacy and tolerability as a therapeutic (58). A similar alteration in GLP-1 receptor signaling profile was recently established for a dual acting GIP-GLP-1 peptide (59). It is thus possible that the observed beneficial effects in vivo of dual GIP-GLP-1 agonists—at least partly—rely on altered signaling of the molecule toward a biased signaling profile for one, or both, of the components. If both mechanisms apply to the GIP- GLP-1 co-agonists, the effect might be even greater. It is still unclear how the GIP part of the co-agonist would lead to weight loss, but if the incorporation of GIP activity in the co-agonist changes the GLP-1 signaling, then it would make sense that even the GIP part of the molecule might contribute to an enhanced weight-losing effect. It should be possible with careful molecular pharmacological experimentation to determine whether it is the influence of one part of the co-agonist (GIP) on the signaling pathways of the other part that makes a co-agonist like tirzepatide so effective, despite the overwhelming evidence that GIP, investigated in isolation, does not possess these activities. Such experiments are ongoing, and we will probably soon have at least some answers to this mind-boggling paradox.

## Additional Information

***Disclosure Summary:*** JJH and MMR are co-founders of Antag Therapeutics and Beinan Biotech. JJH serves on advisory boards for NovoNordisk.

***Data Availability:*** Data sharing is not applicable to this article as no datasets were generated or analyzedduring the current study.
